# Predictive value of triglycerides to high density lipoprotein ratio in patients with first attack of acute coronary syndrome

**DOI:** 10.15537/smj.2023.44.4.20220928

**Published:** 2023-04

**Authors:** Abdullah S. Basuliman, Mohammed A. Malabarey, Fahad W. Abousamak, Bader Y. Alyousef, Saleh S. Alrabea, Rakan A. Alshabibi, Zohair A. Al Aseri

**Affiliations:** *From the College of Medicine (Basuliman, Alyousef, Alrabea), King Saud bin Abdulaziz University for Health Sciences; from the Departments of Emergency Medicine and Critical Care (Malabarey, Al Aseri), College of Medicine, King Saud University; from the Department of Clinical Sciences (Al Aseri), and from the College of Medicine (Abousamak, Alshabibi), Dar Al Uloom University; and from the Saudi Red Crescent Authority (Abousamak), Riyadh, Kingdom of Saudi Arabia.*

**Keywords:** acute coronary syndrome, STEMI, NSTEMI, angina, TG/HDL-C ratio

## Abstract

**Objectives:**

To identify patients who are at risk for a first cardiovascular event, mitigate the risk, and institute early intervention. The triglyceride to high-density lipoprotein-C (TG/HDL-C) ratio has been found to be a very useful biomarker for directing treatment and prevention therapy.

**Methods:**

This retrospective cross-sectional study included adult patients (aged >18 years) experiencing first-time acute coronary syndrome (ACS). We examined all patient databases for a definite diagnosis of angina, non-ST segment elevation myocardial infarction (NSTEMI), or ST-segment elevation myocardial infarction (STEMI). Lipid profiles were obtained prior to or at the time of admission.

**Results:**

A total of 265 patients were included in the study (mean age 57.83 ± 11.4 years) and 79.2% were men. Male gender, presence of diabetes, raised total cholesterol, raised low-density lipoprotein (LDL), and raised troponin level on admission were significantly positively correlated with STEMI (*p*=0.004, *p*=0.001, *p*<0.001, and *p*<0.001), whereas TG/HDL-C ratio was significantly negatively correlated with STEMI (*p*=0.048), while there was no significant results with NSTEMI (*p*=0.264) and angina (*p*=0.326). Total cholesterol and raised low-density lipoprotein (LDL) were significantly positively correlated with NSTEMI (*p*=0.013 and *p*=0.024).

**Conclusion:**

Patients with first-time ACS may not have an increased TG/HDL-C ratio. High LDL levels had the most significant association with an ACS event, along with total cholesterol and diabetes. Further research is needed on a larger scale to determine the association of TG/HDL-C ratio with ACS and differentiate the different types of ACS events according to their clinical and laboratory characteristics.

**A**n estimated 17.9 million people died from cardiovascular diseases (CVDs) in 2019, accounting for 32% of all fatalities worldwide. Heart attacks and strokes accounted for 85% of these.^1^ Cardiovascular diseases have been linked by medical professionals to factors such as high blood pressure, smoking, obesity, inactivity, and high blood cholesterol levels.^1^

A precise estimation of the absolute risk for a first CVD event can help identify patients who need CVD risk assessment and early intervention. According to a study, the triglyceride to high-density lipoprotein (TG/HDL-C) ratio has the strongest correlation with CVD risk and can be a very useful tool for directing treatment and prevention therapy.^2^ In individuals with non-diabetic stable angina pectoris, a significant positive association between the TG/HDL-C ratio and the severity of coronary lesions has been identified. This ratio has also been proposed as a potent independent predictor of all-cause mortality and cardiovascular events.^3,4^ Elevated TG/HDL-C ratio was an independent predictor of long-term all-cause mortality with an increased risk of major adverse cardiac events.^3-5^ In another study, the TG/HDL-C ratio served as a predictor for phenotype B low-density lipoprotein cholesterol (LDL-C) with 79% accuracy.^6^

A survival analysis study also showed that the TG/HDL-C ratio predicted postmenopausal women’s chance of developing carotid plaques, coronary heart disease (CHD), CVD mortality, metabolic syndrome, and diabetes mellitus. In post–percutaneous coronary intervention (PCI) patients with coronary artery disease (CAD), an elevated TG/HDL ratio was also an independent predictor of long-term all-cause mortality of heart failure.^7^ Numerous other studies have demonstrated the substantial correlation between an elevated TG/HDL-C ratio and an increased risk of arterial stiffness, CVD in diabetic individuals, and serious cardiac events due to non-obstructive CAD in women.^8-11^ Several investigations have reported cut-off values for the TG/HDL-C ratio. One study indicated a cut-off of >2.5 in women and >3.5 in men, whereas another reported a ratio cut-off of 3.5 to predict CHD and CVD mortality.^12,13^

We carried out this study to determine the association of TG/HDL-C ratio with acute coronary syndrome (ACS) and differentiate different types of ACS events according to their clinical and laboratory characteristics.

## Methods

This retrospective cross-sectional study with a consecutive sampling technique was carried out at King Saud University Medical City, Riyadh, Saudi Arabia. The study included patients who were older than 18 years with first-time ACS. It used data from the time of admission or the period before the ACS incident (within 6 months) if available. The study excluded patients with hereditary hyperlipidemia, previous diagnosis of ischemic heart disease, inadequate laboratory data, a lack of prior outcomes, and genetic heart issues. We examined all patient notes using the hospital’s electronic health record systems (eSIHI) to check for inclusion, exclusion, and a definite diagnosis of angina, non-ST segment elevation myocardial infarction (NSTEMI) or ST-segment elevation myocardial infarction (STEMI).

**Figure 1:**
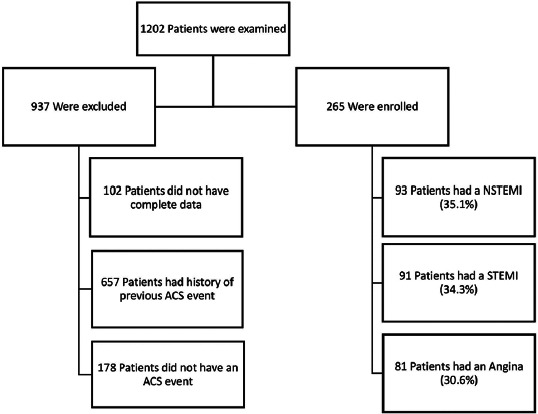
- Flowchart demonstrating sample selection process

The sample size was calculated using the formula n=Z 2P(l—P)/d2, where n is the sample size, Z is the statistic corresponding to the level of confidence, P is the expected prevalence, and d is precision. Assuming that 50% of patients with cardiac events have high TG/HDL-C and using an M precision of 5% and a confidence level of 95%, the calculated sample size was 370 patients. However, due to the limited number of patients who met the criteria, we could only examine 265 patients’ lipid profiles between 2015 and 2021. The process of sample selection is shown in Figure 1.

The TG/HDL ratio (expressed in mmol/L) did not have a definite cut-off value that could be used to determine whether it was high or low. However, according to a recent study from Brazil, a gender-specific cut-off (men: 2.6; women: 1.7) can be used for multiethnic populations with high reliability.^14^ To ascertain the TG/HDL ratio levels in the study population, we adopted this cut-off.

### Statistical analyses

Data analysis were performed using IBM SPSS Statistics for Windows, version 23.0 (IBM-SPSS Inc. Armonk, New York, USA). Variables of interest were expressed as mean and standard deviation for continuous variables and numbers or frequency in percentage for categorical variables. Analysis of variance (ANOVA) was used to compare ACS events with independent variables (such as gender, diabetes, revascularization, total cholesterol, HDL, LDL, triglycerides, TG/HDL, TC/HDL, LDL/HDL, and troponin level on admission). A *p*-value of ≤0.05 was considered statistically significant. Approval for the study protocol was obtained from the Institutional Review Board of King Saud University Medical City.

## Results

A total of 265 patients were included in the study: 210 (79.2%) men and 55 (20.8%) women. The mean age was 57.83 ± 11.4 years. There were 91 (34.3%) with STEMI, 93 (35.1%) with NSTEMI, and 81 (30.6%) with angina. Table 1 shows the detailed demographic and laboratory profile of all 265 patients with ACS.

**Table 1:** - Demographic and laboratory results for all 265 patients with acute coronary syndrome.

Characteristics	n (%)	Mean (SD)
***Gender***		
Male	210 (79.2)	
Female	55 (20.8)	
Age in years		57.83 (11.4)
Total cholesterol		4.60 (1.3)
HDL		1.08 (0.3)
LDL		2.72 (1.1)
Triglycerides		1.80 (1.1)
TG/HDL		1.95 (1.8)
TC/HDL		4.61 (2.2)
LDL/HDL		2.74 (1.8)
Troponin level		5349.87 (9691)
***Troponin***		
>40,000	55 (20.8)	
40 - 40,000	133 (50.2)	
<40	77 (29.1)	
Diabetes, yes	161 (60.8)	

HDL: high-density lipoprotein, LDL: low-density lipoprotein, TG: triglyceride, TC: total-cholesterol

Table 2 shows the proportion of gender, diabetes, and revascularization according to event types. A significantly greater proportion of men had STEMI, whereas women had a greater proportion of angina compared to men (*p*=0.001). A greater proportion of men had diabetes compared to women (*p*=0.017). PCI was more employed with STEMI patients, whereas angina patients were managed more medically (*p*<0.001).

**Table 2:** - The proportion of gender, diabetes, and revascularization according to event types.

Characteristics	STEMI n=91	NSTEMI n=93	Angina n=81	*P*-values
***Gender***				
Male	81 (38.6)	76 (36.2)	53 (25.2)	0.001
Female	10 (18.2)	17 (30.9)	28 (50.9)	
***Diabetic***				
Yes	43 (26.7)	62 (38.5)	56 (34.8)	0.017
No	48 (46.2)	31 (29.8)	25 (24.0)	
***Revascularization***				
Medical treatment	8 (14.5)	18 (32.7)	29 (52.7)	
PCI	68 (54.4)	41 (32.8)	16 (12.8)	
CABG	10 (23.3)	19 (44.2)	14 (32.6)	<0.001
Thrombolytics	2 (100.0)	0	0	
None	3 (7.5)	15 (37.5)	22 (55.0)	

Values are presented a number and percentages (%). NSEMI: non-ST segment elevation myocardial infarction, STEMI: ST-segment elevation myocardial infarction, PCI: percutaneous coronary intervention, CABG: coronary artery bypass graft

Correlation analysis showed that male gender, presence of diabetes, raised total cholesterol, raised LDL, and raised troponin level on admission were significantly positively correlated with STEMI (*p*=0.004, *p*=0.001, *p*<0.001, and *p*<0.001), whereas TG/HDL-C ratio was significantly negatively correlated with STEMI (*p*=0.048) and revascularization (*p*=0.016). Triglyceride to high-density lipoprotein-C ratio did not show a significant relationship with either NSTEMI (0.264) or angina (0.326). Our study revealed that the average TG/HDL-C ratio was 1.79 for men and 1.77 for women. Individuals with diabetes had a mean TG/HDL-C ratio of 2.09 compared to 1.72 in nondiabetic patients. Total cholesterol and raised LDL were significantly positively correlated with NSTEMI (*p*=0.013 and *p*=0.024), whereas HDL was significantly negatively correlated with NSTEMI (*p*=0.029). Angina was significantly positively correlated with raised LDL and raised triglycerides (*p*=0.005 and *p*=0.043) but significantly negatively correlated with male gender and troponin level on admission (*p*<0.001 and *p*=0.001; Table 3).

**Table 3:** - The proportion of gender, diabetes, and revascularization according to event types.

Variables	STEMI n=91 Correlation (p)	NSTEMI n=93 Correlation (p)	Angina n=81 Correlation (p)
Age, mean (SD)	-0.079 (0.199)	0.109 (0.076)	-0.031 (0.611)
Gender, male	0.174 (0.004)*	0.045 (0.467)	-0.225 (<0.001)*
Diabetes	0.207 (0.001)*	0.093 (0.131)	0.119 (0.053)
Total cholesterol	0.245 (<0.001)*	0.153 (0.013)*	0.094 (0.128)
HDL	0.042 (0.498)	-0.134 (0.029)*	0.093 (0.130)
LDL	0.306 (<0.001)*	0.139 (0.024)*	0.173 (0.005)*
Triglycerides	-0.116 (0.059)	0.004 (0.949)	0.125 (0.043)*
TG/HDL	-0.122 (0.048)*	0.069 (0.264)	0.061 (0.326)
TC/HDL	0.069 (0.260)	-0.021 (0.731)	-0.048 (0.440)
LDL/HDL	0.136 (0.027)	-0.056 (0.366)	-0.083 (0.179)
Troponin level on admission	0.368 (<0.001)*	-0.104 (0.179)	-0.255 (0.001)*
Revascularization	-0.148 (0.016)*	0.042 (0.495)	0.109 (0.078)

Values are presented a number and percentages (%). NSEMI: non-ST segment elevation myocardial infarction, STEMI: ST-segment elevation myocardial infarction,*significant, HDL: high-density lipoprotein, LDL: low-density lipoprotein, TG: triglyceride, TC: total-cholesterol

## Discussion

Obesity, waist-to-hip ratio, abdominal waist circumference, and body mass index are just a few of the metrics with significant predictive values for cardiovascular events that have been reported, although many of these studies produced contradictory findings.^15-17^ Despite the current standards of care for secondary prevention, which include lifestyle changes, ideal medical therapy, myocardial revascularization, and the use of antiplatelet medications to reduce thrombosis, the risks of cardiovascular events are commonly overestimated and persist. In this investigation, we examined patients who had ACS for the first time, and we identified the clinical and laboratory characteristics of the various types of ACS occurrences. The inclusion of only patients with their first ACS incident was a key component of our investigation. Since having a prior myocardial infarction can raise the likelihood of a second myocardial infarction or stroke, this guarantees that other risk factors have the least impact possible.

We discovered that men were more likely than women to experience STEMI. This is comparable to the study by George et al^18^ that showed a male preponderance for STEMI in 2021. Similar results demonstrating a higher prevalence of STEMI in men than women have been reported in several other investigations.^19,20^ On the other hand, we discovered that women had a higher prevalence of angina than men. This is consistent with prior information that some traditional risk factors, such as diabetes, hypertension, hypercholesterolemia, and obesity, increase the risk of CVD in women more than in men, and that socioeconomic and psychosocial factors may also have a greater effect on CVD in women.^21^ Although women are older and have more comorbid conditions than men, a gender bias may exist in favor of men concerning variations in CVD care: 50% of men underwent PCI compared to 36.4% of women, which could lead to a poorer outcome.

We also discovered a substantial correlation between STEMI and diabetes, hypercholesterolemia, and high LDL levels. This is in line with other research showing that elevated residual cholesterol raises the risk of peripheral arterial disease in the general population by five times, surpassing that of myocardial infarction and ischemic stroke.^22-24^ As our study also revealed, the incidence of composite clinical events has also been shown to significantly increase as the admission troponin levels increased.^25^

The TG/HDL ratio had an unusual inverted relationship with STEMI, with men having a mean of 1.68 and women having a mean of 1.3. This can be attributed to the lipid paradox, where the presence of atherosclerotic changes and inflammatory processes due to medical conditions, medications, and lifestyle factors can be behind these unusual results with STEMI compared to NSTEMI and angina.^26^ The mean value for NSTEMI was 2.12 and angina was 2.09. In contrast, an Iranian study^27^ found that a mean TG/HDL-C cut-off of 3.5 was appropriate for both men and women and had good sensitivity and specificity. When compared to the low-risk group, the TG/HDL ratio was significantly higher in patients with a high CVD risk.^27^ For a clearer understanding of the TG/HDL-C ratio, we recommend further research in this area.

Among all ACS occurrences in this study, LDL showed the highest association. In the non-diabetic group, the mean LDL level was 2.96 compared to 2.57 in the diabetes group. The diabetic angina group had the lowest mean LDL level (2.26), while the non-diabetic STEMI group had the highest (3.35). Similar patterns existed in the mean total cholesterol levels, with the diabetic angina group having the lowest levels (4.31) and the non-diabetic STEMI group having the highest (5.16). According to the literature, including the Framingham study, this is valid.^28^ Diabetes patients have a known increased risk of developing ACS. Atherosclerosis and the development of plaques that cause adverse cardiac events are primarily induced by hyperglycemia, insulin resistance, and vascular calcification.^29^ This is significant since, according to a 2017 report, the prevalence of diabetes in Saudi Arabia may exceed 30%.^30^ This prevalence is high and rising among the population of the country.

### Study limitations

This investigation has a few limitations. Relationships do not necessarily imply causation in this cross-sectional study design. Since the majority of patients did not satisfy the research’s eligibility requirements, we had trouble obtaining the appropriate sample for our study. This was primarily the result of data that either was inadequately reported or included a history of ischemic heart disease. Another drawback is the lack of a clearly defined cut-off value for the Saudi population, which is necessary due to variations in TG/HDL levels between various locations. We advise conducting a study with a sufficient sample size for the Saudi population to establish a reasonable cut-off.

In conclusion, patients with first-time ACS might not have increased TG/HDL-C ratios. The strongest correlation was found between ACS occurrence and high LDL levels, total cholesterol, and diabetes. A more extensive study is needed to establish the relationship between the TG/HDL-C ratio and ACS and to distinguish the various ACS occurrences based on their clinical and laboratory features.
